# Fatigue, symptom burden, and health‐related quality of life in patients with myelodysplastic syndrome, aplastic anemia, and paroxysmal nocturnal hemoglobinuria

**DOI:** 10.1002/cam4.1953

**Published:** 2019-01-11

**Authors:** Carmen P. Escalante, Stephanie Chisolm, Juhee Song, Marsha Richardson, Ellen Salkeld, Etsuko Aoki, Guillermo Garcia‐Manero

**Affiliations:** ^1^ Department of General Internal Medicine The University of Texas MD Anderson Cancer Center Houston Texas; ^2^ Bladder Cancer Advocacy Network Bethesda Maryland; ^3^ Department of Biostatistics The University of Texas MD Anderson Cancer Center Houston Texas; ^4^ Aplastic Anemia and MDS International Foundation Bethesda Maryland; ^5^ Department of Leukemia The University of Texas MD Anderson Cancer Center Houston Texas

**Keywords:** aplastic anemia, fatigue, myelodysplastic syndrome, paroxysmal nocturnal hematuria, quality of life

## Abstract

**Background:**

Fatigue is distressing and affects quality of life (QoL) among patients with myelodysplastic syndrome (MDS), aplastic anemia (AA), and paroxysmal nocturnal hemoglobinuria (PNH). Limited data exist on the impact of fatigue, QoL, and related symptoms in these patients.

**Objective:**

Prospectively assess fatigue (functional assessment of cancer therapy‐anemia [FACT‐An]); QoL (FACT‐An subscales); pain (brief pain inventory); and depression, anxiety, and stress (depression anxiety stress scale‐21) and strategies used to manage these symptoms in patients with MDS, AA, and PNH.

**Methods:**

Surveys were administered via the AA and MDS International Foundation website and database from October 2014 through April 2015 in a cross‐sectional study. Results were summarized using descriptive statistics.

**Results:**

Of 303 patients, 145 (48%) had MDS, 84 (28%) had AA, and 74 (24%) had PNH; 31 (10%) had >1 diagnosis. The mean age was 57 years, 200 (66%) were female, and 195 (92%) were white. The mean fatigue scores were 25 (range 1‐52) for the whole cohort, 28 for AA, 25 for MDS, and 24 for PNH (*P* = 0.117); these are all considered severe level. The mean QoL score was 68 (range 10‐104) for the whole cohort, 67 for AA, 69 for MDS, and 67 for PNH (*P* = 0.821). The ranges for stress were normal; pain and depression, mild; and anxiety, moderate. The most common management strategies perceived as helpful for fatigue in the past month were preserving energy, physical activity, and naps.

**Conclusions:**

Many patients with MDS, AA, and PNH report severe fatigue. The helpfulness of fatigue management strategies may impact patients’ continued use; whether these strategies are beneficial and decrease fatigue levels needs more study.

## 1 INTRODUCTION

Myelodysplastic syndrome (MDS), aplastic anemia (AA), and paroxysmal nocturnal hemoglobinuria (PNH) are rare disorders of bone marrow failure and have overlapping pathophysiologies.^1^ AA and PNH are not defined as malignancies but may transition to leukemias.^2^ However, the American Cancer Society now considers MDS a malignancy.

Myelodysplastic syndrome includes a diverse group of hematopoietic stem cell disorders categorized by dysplastic and ineffectual blood cell production. The incidence is approximately 3‐4 cases/100 000; an estimated 10 000 cases are diagnosed annually in the United States.^3^, ^4^, ^5^ The actual incidence may be higher owing to often nonspecific symptoms, and definitive diagnosis with appropriate testing may not occur. Symptoms may be nonspecific, but anemia is the most common cytopenia observed and is frequently associated with fatigue, weakness, exercise intolerance, angina, or cognitive impairment.^6^, ^7^, ^8^, ^9^

Aplastic anemia is a rare disease featuring decreased or absent hematopoietic precursors in the bone marrow, often secondary to pluripotent stem cell injury.^10^, ^11^ Environmental exposures such as benzene and pharmaceutical drug use with chloramphenicol were initially reported as culprits leading to AA. Frequently, patients present with fatigue and symptoms associated with anemia such as pallor, headache, dyspnea, palpitations, gingival bleeding, and petechial rashes. The incidence varies throughout the world. Montane et al reported an incidence of 2.34 per million in Barcelona, and similar rates of 2.0 per million were reported by the International Agranulocytosis and Aplastic Anemia Study in Europe and Israel.^10^, ^12^

Paroxysmal nocturnal hemoglobinuria is another rare acquired hematopoietic stem cell disorder with nonspecific clinical features that often delay diagnosis. The incidence ranges from 1 to 10 cases per million but is likely underestimated because some patients may remain undiagnosed.^13^, ^14^ PNH has a median age of onset in the thirties^15^, ^16^ but may occur in childhood, although less frequently.^17^, ^18^ The most prevalent symptoms are fatigue (80%), dyspnea (64%), and hemoglobinuria (62%).^15^

As noted, fatigue is a very common and often the most frequent symptom among patients with these very rare hematopoietic stem cell disorders, and it is very distressing for patients.^19^ Fatigue may significantly affect patients’ quality of life (QoL) and productivity.^19^ Because it is very difficult to study rare diseases owing to their infrequency and fatigue is often a leading symptom in these diseases, we approached MDS, AA, and PNH as a combined entity so that we would have a larger group to study, understanding the limitations arising from this approach. Although fatigue is prevalent in these diseases, more specific information about this symptom related to these stem cell disorders is limited and not well studied.

In addition, studies of cancer‐related fatigue have shown that fatigue exists as a cluster of symptoms, including pain and depression. Multiple factors may contribute to fatigue, so the treatment and resolution of these factors may reduce or even resolve the symptom of fatigue.^20^ We assumed that this would be the case in these diseases we were studying. Furthermore, understanding of the impact of MDS, AA, and PNH on an individual's QoL is limited. Traditionally, physicians have targeted the physical aspects of MDS such as improving cytopenias and preventing evolution to leukemia.^21^ However, in recent years, clinicians have been focusing on the QoL of MDS patients.^21^ Many of the survey tools used in the literature to measure the health‐related QoL of MDS patients are instruments used in cancer studies.^19^, ^22^, ^23^, ^24^ Currently, there is little data assessing the impact of fatigue on patients’ QoL in these diseases, especially in AA and PNH. Finally, management strategies and the patient's perception of the helpfulness of these strategies have not been well studied in these populations and are important in understanding appropriate treatment approaches.

Therefore, the purpose of our study was (a) to learn about fatigue and the associated symptom burden (pain, depression, anxiety, stress); (b) to assess QoL using the Functional Assessment of Cancer Therapy‐Anemia (FACT‐An); and (c) to identify management strategies patients use routinely and whether they are perceived as helpful to patients with MDS, AA, and PNH.

## 2 METHODS

This observational, descriptive cross‐sectional study was approved by The University of Texas MD Anderson Cancer Center's Institutional Review Board. In collaboration with the Aplastic Anemia and MDS International Foundation, Survey Monkey (SurveyMonkey Inc.; Palo Alto, CA, USA; www.surveymonkey.com) was employed to achieve the study's objectives by administering surveys to patients with MDS, AA, and PNH registered in the Foundation's database. The Foundation focuses their work on these three rare hematologic disorders. Survey Monkey is a commercial entity that can electronically distribute survey materials with proper safeguards of confidentiality, including Secure Sockets Layer encryption. It was initially developed for transmitting private documents or information via the internet and essentially works through a cryptographic system that secures a connection between a client and a server. The surveys were housed on the Foundation's secured server.

### 2.1 Design

The Foundation sent an invitation as an e‐Blast to their entire patient database of approximately 17 500 patients, although it is not known when or how frequently these patients interacted with the website. Patients accessing the website and choosing to participate were linked to the study's landing page containing a consent statement. All patients visiting the website had an opportunity to participate in the study and complete the surveys. The following text concluded the consent statement: “If you understand and consent to participate, please click here to begin.” The goal was to reach a heterogeneous group of patients with these rare diseases.

Following the initial e‐Blast, we sent reminders to the Foundation's patient database via the Foundation's electronic newsletters and research newsletters in December 2014, as well as twice in January 2015 and once in February 2015. Facebook and Twitter announcements regarding the survey were sent via the Foundation in January 2015. These announcements included all patients, both those who may have already completed the survey and those who may not have completed the survey.

The survey was completed by patients who visited the website of the Aplastic Anemia and MDS International Foundation during this timeframe and met the following inclusion criteria: 18 years or older; diagnosis of MDS, AA, or PNH; able to complete the required survey tools independently; and able to read and write in English. Not all patients completed every item in the survey. There were no automatic stops built into the surveys if patients did not complete an item.

### 2.2 Sample size

**Figure 1:**
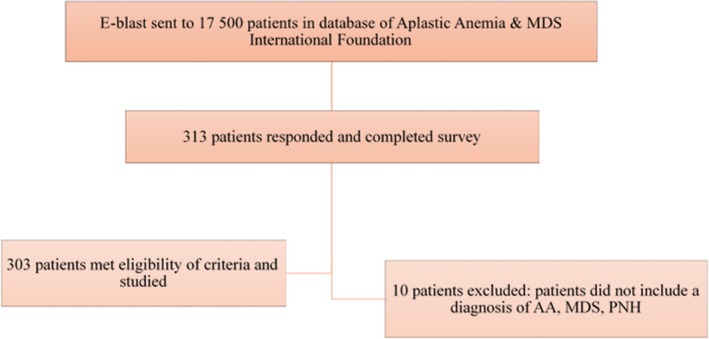
A total of 303 patients were studied

A total of 313 patients enrolled in this observational study from December 15, 2014, through April 13, 2015, through the Foundation's website; however, 10 patients did not include a diagnosis of one of these diseases. Therefore, 303 patients met eligibility criteria (Figure 1).

### 2.3 Survey content

The survey included the following aspects: demographic information (age, sex, race, and country of residency); social information (education, marital status, employment status); clinical parameters (date of diagnosis, type of disease, past and present treatments, and use and frequency of various types of management for fatigue and perception of helpfulness). Patients also completed the functional assessment of cancer therapy‐anemia (FACT‐An),^25^ the brief pain inventory (BPI),^26^ and the depression anxiety stress scale (DASS 21).^27^ The types of fatigue management included physical activity, prescription stimulants, over‐the‐counter vitamins, over‐the‐counter herbs, over‐the‐counter minerals, naps, preserving energy, acupuncture, meditation, eating healthy, and counseling/support groups. Study participants indicated how often they use these tools (never use, 1‐2 times weekly, 3‐4 times weekly, and 5 or more times weekly). Participants also rated the perceived helpfulness of these tools over the past month from 1 (not helpful) to 5 (extremely helpful).

### 2.4 Instruments used

The FACT‐An consists of the Functional Assessment of Cancer Therapy‐Fatigue (FACT‐F), which includes the Functional Assessment of Cancer Therapy‐General (FACT‐G) instrument measuring general QoL as a core questionnaire plus 13 fatigue items, as well as an additional seven items related to anemia but unrelated to fatigue. Therefore, the FACT‐An contains a total of 47 items. Both the FACT‐F and the FACT‐An demonstrate good stability (*r* = 0.87 for both) and strong internal consistency (*α* = 0.95 for FACT‐F and 0.96 for FACT‐An).^25^ Higher scores indicate better QoL.

The BPI^26^ assesses the severity of pain and its impact on functioning. The BPI assesses pain at its “worst,” “least,” “average,” and “now.” The BPI has a Cronbach alpha reliability ranging from 0.77 to 0.91. The BPI Short Form consists of nine items. Scores of <4 are rated as mild pain, scores of 4 through 6.9 are moderate pain, and scores of 7 or higher are severe pain.

The DASS 21^27^ consists of three self‐report scales with 21 total items designed to measure the negative emotional states of depression, anxiety, and stress. The depression scale assesses dysphoria, hopelessness, devaluation of life, self‐deprecation and lack of interest/involvement, anhedonia, and inertia. The anxiety scale assesses difficulty relaxing, nervous arousal, and being easily upset/agitated, irritable/over‐reactive, and impatient. The DASS 21 psychometric properties have excellent internal consistency and temporal stability.^28^ Scores for depression are as follows: normal 0‐9, mild 10‐13, moderate 14‐20, severe 21‐27, and extremely severe 28 or higher. Scores for anxiety are normal 0‐7, mild 8‐9, moderate 10‐14, severe 15‐19, and extremely severe 20 or higher. Stress scores are normal 0‐14, mild 15‐18, moderate 19‐25, severe 26‐33, and extremely severe 34 or higher.

### 2.5 Primary outcomes

Primary endpoints were the FACT‐An fatigue subscale score (range 0‐52)^25^; DASS 21 subscale scores, including depression (range 0‐42), anxiety (range 0‐42), and stress scores (range 0‐42)^27^; and BPI score (range 0‐10)^26^. Other endpoints included continuous variables (FACT‐An total score, FACT‐An non‐fatigue subscale score, and other subscale scores) and categorical variables (use of each fatigue management tool and perception of the helpfulness of each fatigue management tool).

### 2.6 Statistics

This was a descriptive study. Patient demographic and other disease‐specific characteristics were analyzed using descriptive statistics, including mean (standard deviation) median (range) for continuous variables and frequency (percentage) for categorical variables. FACT‐An^25^, BPI^26^, and DASS 21^27^ were scored using appropriate scoring algorithms. Missing values were handled as recommended by the developers of the instruments. Subgroups (normal, mild, moderate, severe, extremely severe) were defined in terms of depression, anxiety, and stress using cutoff scores for defining degrees of severity proposed by developers of DASS 21.^27^

Fatigue (FACT‐An fatigue subscale score) and other symptom burden (DASS 21 total score, depression score, anxiety score, and stress score; subgroups in terms of depression, anxiety, and stress; BPI pain severity index and function interference index) and QoL (FACT‐An total score, FACT‐An non‐fatigue subscale score, and other subscale scores) were summarized using means (standard deviation) and medians (range) or frequency (percentage) in all patients and by disease group. Three disease groups were compared using the chi‐square test, Fisher exact test, analysis of variance, or Kruskal‐Wallis test, depending on the specific comparison and characteristics of the data distribution. The proportion of patients using each fatigue management tool and the 95% confidence interval were estimated. The perception of helpfulness of each fatigue management tool was summarized using means (standard deviation) and medians (range) or frequency (percentage). A *P* value of <0.05 indicated statistical significance. SAS 9.3 (SAS Institute INC, Cary, NC) was used for data analysis.

## 3 RESULTS

### 3.1 Demographics

Of 303 patients, 145 (48%) had MDS (MDS only), 84 (28%) had AA (AA only or AA/MDS), and 74 (24%) had PNH (PNH only, PNH/AA, MDS/PNH, MDS/AA/PNH). Thirty‐one patients (10%) had more than one of these diagnoses. The mean age was 57 years, 200 (66%) were female, and nearly all were white (92% of 211 with known race). Patients with MDS were the oldest (mean 67 years) and those with PNH were the youngest (mean 44 years). The mean age of the AA patients was 51 years. Significant differences were detected in mean ages between the three diagnoses (*P* < 0.0001). Furthermore, a higher proportion of men had MDS (43%) than AA (30%) or PNH (22%; *P* = 0.005; Table 1).

**Table 1:** Demographic characteristics of study participants (n = 303)^a^

Variable	No. (%)				*P*
	Overall	MDS, n = 145	AA, n = 84	PNH, n = 74	
Mean ± SD age (range)	57 ± 16 y	67 ± 8 y	51 ± 15 y	44 ± 15 y	0.0001
	(18‐90 y)	(47‐90 y)	(19‐88 y)	(18‐76 y)	
Sex	303 (100)				0.005
Female	200 (66)	83 (42)	59 (30)	58 (29)	
Race	211 (70)				0.105
White	195 (92)	92 (47)	48 (25)	55 (28)	
Education	216 (71)				0.479
Graduate degree	74 (34)	42 (57)	15 (20)	17 (23)	
College degree/some college	122 (56)	47 (39)	35 (29)	40 (33)	
High school degree	18 (9)	8 (44)	5 (28)	5 (28)	
Less than high school degree	2 (1)	1 (50)	1 (50)	0	
Employment status	216 (71)				<0.001
Working full time	69 (32)	19 (28)	23 (33)	27 (39)	
Working part time	25 (12)	6 (24)	10 (40)	9 (36)	
Unemployed	25 (12)	4 (16)	10 (40)	11 (44)	
Disabled	28 (13)	12 (43)	6 (21)	10 (36)	
Retired	69 (32)	57 (83)	7 (10)	5 (7)	
Marital status	216 (70)				0.003
Married	147 (68)	76 (52)	35 (24)	36 (24)	
Partner	14 (6)	3 (21)	2 (14)	9 (64)	
Single	28 (13)	4 (14)	13 (46)	11 (39)	
Divorced/separated/widow	27 (13)	16 (59)	6 (22)	5 (19)	

AA, aplastic anemia; MDS, myelodysplastic syndrome; PNH, paroxysmal nocturnal hemoglobinuria.

a Age was known for 302 patients. For other variables, percentages reflect the number of patients in each group for which the information was known.

### 3.2 Clinical characteristics

The mean time from diagnosis of MDS, AA, or PNH to the time of the survey was 7.6 years for the overall group (Table 2). Approximately 19% of patients had received a red blood cell transfusion in the previous 90 days. In addition, 11% (31 of 280 patients responding) had received a prior stem cell transplantation; this variable did not differ among the subgroups (*P* = 0.549). Six patients had undergone a stem cell transplantation in the past year (two from each subgroup), three in the past 90 days (one with AA and two with MDS). Sixty‐four patients (24%) had received growth factors in the past 90 days, although only 10 (4%) had received iron chelation therapy in the past 90 days. Approximately 45% of patients (122 of 274 responding) had not received any medical treatment or drug therapy for their disease in the past 90 days. Thirty‐nine patients (15%) did not participate in regular exercise.

**Table 2:** Clinical characteristics of study participants (n = 303)^a^

Factor	No. (%)				*P*
	Overall	MDS, n = 145	AA, n = 84	PNH, n = 74	
Mean time from diagnosis	7.6 y	6.6 y	7.1 y	9.8 y	<0.001
RBC transfusion in past 90 d	53 (19)	30 (57)	13 (25)	10 (19)	0.320
Platelet transfusion in past 90 d	30 (11)	13 (43)	13 (43)	4 (13)	0.075
Received growth factors in past 90 d	64 (24)	41 (64)	14 (22)	9 (14)	0.011
Received erythropoietin in past 90 d	41 (15)	32 (78)	6 (15)	3 (7)	<0.001
Received granulocyte‐colony stimulating factor in past 90 d	18 (7)	9 (50)	3 (17)	6 (33)	<0.001
Received granulocyte‐macrophage stimulating factor in past 90 d	2	2 (100)	0	0	0.338
Received oprelvekin in past 90 d	2	2 (100)	0	0	0.338
Previous stem cell transplantation	31 (11)	14 (45)	11 (35)	6 (19)	0.549
Received iron transfusion in past 90 d	10 (4)	7 (70)	1 (10)	2 (20)	0.313
Received no medical/drug therapy in past 90 d	122 (45)	74 (61)	34 (28)	14 (11)	<0.001
Exercise frequency	260 (86)	125 (48)	70 (27)	65 (25)	0.033
No regular exercise	39 (15)	25 (64)	2 (5)	12 (31)	
1‐2 d/wk	95 (37)	41 (43)	30 (32)	24 (25)	
3‐4 d/wk	68 (26)	33 (49)	17 (25)	18 (26)	
5‐7 d/wk	58 (22)	26 (45)	21 (36)	11 (19)	

AA, aplastic anemia; MDS, myelodysplastic syndrome; PNH, paroxysmal nocturnal hemoglobinuria; RBC, red blood cell.

a Not all patients answered each question.

### 3.3 Symptom measurement

Fatigue, QoL, pain, depression, anxiety, and stress were not significantly different among patients with AA, MDS, and PNH (Table 3). Fatigue was in a severe range, QoL was decreased, pain was mild, depression was mild, anxiety was moderate, and stress was in normal range for all subgroups. The physical, social/family, emotional, and functional well‐being items from the Fact‐An^25^ did not differ among the subgroups. Stress was normal in all subgroups, pain and depression were mild in all subgroups, and anxiety was mild in patients with MDS and moderate in those with AA and PNH. No significant differences were observed among the subgroups for any of these symptoms, including anxiety.

**Table 3:** Fatigue and other symptoms overall and by group

Survey tool	Overall mean (range)	Overall severity level	Mean (range)			*P*
			MDS, n = 145	AA, n = 84	PNH, n = 74	
FACT‐F, n = 258	25 (1‐52)	Severe	25 (5‐52) severe	28 (1‐52) severe	24 (2‐52) severe	0.117
FACT‐G, n = 252	68 (10‐104)	a	69 (16‐99)	67 (10‐99)	67 (28‐104)	0.821
FACT‐An, n = 252	111 (14‐184)	a	111 (30‐173)	114 (14‐178)	109 (40‐184)	0.540
BPI, n = 259	2 (0‐8)	Mild	2 mild	2 mild	2 mild	0.386
DASS‐Depression, n = 264	12 (0‐42)	Mild	12 (0‐42) mild	13 (0‐42) mild	12 (0‐38) mild	0.972
DASS‐Anxiety, n = 263	10 (0‐42)	Moderate	8 (0‐38) mild	12 (0‐42) moderate	10 (0‐36) moderate	0.067
DASS‐Stress, n = 264	12 (0‐40)	Normal	12 (0‐40) normal	13 (0‐40) normal	13 (0‐36) normal	0.304

AA, aplastic anemia; BPI, brief pain inventory; DASS, depression anxiety stress scale; FACT, functional assessment of cancer therapy; ‐An, anemia; ‐F, fatigue; ‐G, general; MDS, myelodysplastic syndrome; PNH, paroxysmal nocturnal hemoglobinuria.

a The higher the score, the better the quality of life.

### 3.4 Fatigue management strategies used

The most common fatigue management strategies used (at any frequency) among the entire group during the past month were preserving energy, physical activity, and naps, and the least common strategies used were acupuncture, prescription stimulants, and herbal supplements. The overall group reported that they used vitamins (84%), healthy eating (60%), and physical activity (49%) three or more times per week. Strategies most commonly never used were acupuncture (97%), prescription stimulants (93%), and herbal supplements (86%).

### 3.5 Frequency of fatigue management strategies utilized

The frequency of physical activity (*P* = 0.033), healthy eating (*P* = 0.005), and counseling (*P* = 0.005) differed among the subgroups. Those with AA were more likely to use physical activities three or more times per week (54%) than were patients with MDS (47%) or PNH (45%), and fewer patients with AA (3%) never used physical activity compared with patients with MDS (20%) and PNH (19%). For healthy eating, 65% with MDS, 57% with PNH, and 54% with AA practiced this three or more times weekly and 25% with MDS, 15% with AA, and 12% with PNH never practiced this. Counseling was never used by 86% with MDS, 82% with AA, and 67% with PNH (Table 4).

**Table 4:** Fatigue management strategies used^a^

Management strategy	Frequency	No. (%)				*P*
		Overall	MDS, n = 145	AA, n = 84	PNH, n = 74	
Physical activity	1‐2×/wk	95 (37)	41 (43)	30 (32)	24 (25)	0.033
	3‐4×/wk	68 (26)	33 (49)	17 (25)	18 (26)	
	>5×/wk	58 (22)	26 (45)	21 (36)	11 (19)	
	Never	39 (15)	25 (64)	2 (5)	12 (31)	
Prescription stimulants	1‐2×/wk	5 (2)	1 (20)	2 (40)	2 (40)	0.346
	3‐4×/wk	3 (0.3)	0	1 (33)	2 (67)	
	>5×/wk	9 (4)	3 (33)	4 (44)	2 (22)	
	Never	231 (93)	114 (49)	60 (26)	57 (25)	
OTC vitamins	1‐2×/wk	16 (6)	7 (44)	3 (19)	6 (38)	0.848
	3‐4×/wk	24 (9)	10 (42)	8 (33)	6 (25)	
	>5×/wk	113 (44)	58 (51)	28 (25)	27 (24)	
	Never	103 (41)	47 (46)	29 (28)	27 (26)	
OTC herbs	1‐2×/wk	8 (3)	4 (50)	2 (25)	2 (25)	0.997
	3‐4×/wk	9 (4)	4 (44)	3 (33)	2 (22)	
	>5×/wk	17 (7)	9 (53)	4 (24)	4 (24)	
	Never	213 (86)	98 (46)	58 (27)	57 (27)	
OTC minerals	1‐2×/wk	9 (3)	4 (44)	2 (22)	3 (33)	0.945
	3‐4×/wk	22 (9)	10 (45)	6 (27)	6 (27)	
	>5×/wk	74 (30)	39 (53)	19 (26)	16 (22)	
	Never	143 (58)	64 (45)	40 (28)	39 (27)	
Naps	1‐2×/wk	80 (31)	33 (41)	24 (30)	23 (29)	0.597
	3‐4×/wk	62 (24)	27 (44)	18 (29)	17 (27)	
	>5×/wk	66 (25)	33 (50)	19 (29)	14 (21)	
	Never	49 (19)	28 (57)	9 (18)	12 (24)	
Preserving energy	1‐2×/wk	70 (27)	25 (36)	27 (39)	18 (26)	0.052
	3‐4×/wk	69 (27)	36 (52)	16 (23)	17 (25)	
	>5×/wk	93 (36)	44 (47)	21 (23)	28 (30)	
	Never	28 (11)	19 (68)	4 (14)	5 (18)	
Acupuncture	1‐2×/wk	5 (2)	1 (20)	1 (20)	3 (60)	0.506
	3‐4×/wk	1 (0.5)	1 (100)	0	0	
	>5×/wk	1 (0.5)	1 (100)	0	0	
	Never	243 (97)	114 (47)	66 (27)	63 (26)	
Meditation	1‐2×/wk	58 (28)	20 (34)	11 (19)	27 (47)	0.302
	3‐4×/wk	18 (7)	5 (28)	6 (33)	7 (39)	
	>5×/wk	6 (2)	4 (67)	1 (17)	1 (17)	
	Never	183 (72)	90 (49)	52 (28)	41 (22)	
Healthy eating	1‐2×/wk	54 (21)	13 (24)	21 (39)	20 (37)	0.005
	3‐4×/wk	58 (23)	29 (50)	17 (29)	12 (21)	
	>5×/wk	95 (37)	49 (52)	20 (21)	26 (27)	
	Never	48 (19)	30 (63)	10 (20)	8 (17)	
Counseling/support groups	1‐2×/wk	42 (17)	13 (31)	9 (21)	20 (48)	0.005
	3‐4×/wk	4 (2)	0	3 (75)	1 (25)	
	>5×/wk	4 (2)	3 (75)	0	1 (25)	
	Never	198 (80)	99 (50)	55 (28)	44 (22)	

AA, aplastic anemia; MDS, myelodysplastic syndrome; OTC, over the counter; PNH, paroxysmal nocturnal hemoglobinuria.

a Not all patients answered each question.

### 3.6 Fatigue management strategies perceived as helpful

Strategies that were perceived as helpful to extremely helpful (rated 3 through 5) were preserving energy (86%), physical activity (70%), and naps (68%). Strategies felt to be most unhelpful (rated 1 or 2) were acupuncture (42%) and counseling (40%). Strategies that differed among the subgroups (MDS, AA, and PNH) in terms of patients’ perception of helpfulness were physical activity (*P* = 0.029), and meditation (*P* = 0.031). Eighty percent of patients with MDS, 68% with PNH, and 58% with AA perceived physical activity as helpful to extremely helpful. For meditation, 85% of patients with AA, 68% of patients with PNH, and 59% of patients with MDS perceived it as helpful to extremely helpful (Table 5).

**Table 5:** Fatigue management strategies perceived as helpful^a^

Management strategy	Level of helpfulness^b^	No. (%)				*P*
		Overall	MDS, n = 145	AA, n = 84	PNH, n = 74	
Physical activity	1	12 (5)	1 (8)	8 (67)	3 (25)	0.029
	2	57 (25)	20 (35)	21 (37)	16 (28)	
	3	73 (32)	37 (51)	20 (27)	16 (22)	
	4	41 (18)	17 (41)	12 (29)	12 (29)	
	5	46 (20)	27 (59)	8 (17)	11 (24)	
Prescription stimulants	1	3 (11)	0	1 (33)	2 (67)	0.069
	2	7 (26)	2 (29)	3 (42)	2 (29)	
	3	7 (26)	1 (14)	4 (57)	2 (29)	
	4	3 (11)	0	1 (33)	2 (67)	
	5	7 (26)	3 (43)	2 (29)	2 (29)	
OTC vitamins	1	10 (6)	6 (60)	2 (20)	2 (20)	0.393
	2	47 (29)	19 (40)	13 (28)	15 (32)	
	3	67 (37)	27 (40)	29 (43)	11 (16)	
	4	27 (17)	16 (59)	5 (19)	6 (22)	
	5	21 (13)	12 (57)	2 (10)	7 (33)	
OTC herbs	1	6 (15)	4 (67)	1 (17)	1 (17)	0.136
	2	8 (21)	2 (25)	4 (50)	2 (25)	
	3	12 (31)	6 (50)	4 (33)	2 (17)	
	4	5 (13)	1 (20)	0	4 (80)	
	5	8 (21)	5 (63)	1(13)	2 (25)	
OTC minerals	1	10 (8)	5 (50)	3 (30)	2 (20)	0.755
	2	26 (22)	11 (42)	7 (27)	8 (31)	
	3	41 (35)	22 (54)	13 (32)	6 (15)	
	4	24 (21)	13 (54)	5 (21)	6 (25)	
	5	16 (14)	9 (56)	2 (13)	5 (31)	
Naps	1	6 (3)	5 (83)	1 (17)	0	0.360
	2	66 (30)	30 (45)	23 (35)	13 (20)	
	3	56 (25)	22 (39)	15 (27)	19 (34)	
	4	49 (22)	24 (49)	11 (22)	14 (29)	
	5	46 (21)	19 (41)	13 (28)	14 (30)	
Preserving energy	1	1 (0.4)	0	0	1 (100)	0.287
	2	32 (13)	11 (34)	8 (25)	13 (41)	
	3	67 (27)	31 (46)	21 (31)	15 (23)	
	4	84 (34)	42 (50)	25 (30)	17 (20)	
	5	63 (25)	30 (48)	14 (22)	19 (30)	
Acupuncture	1	2 (12)	1 (50)	1 (50)	0	0.823
	2	5 (30)	1 (20)	2 (40)	2 (40)	
	3	5 (30)	2 (40)	1 (20)	2 (40)	
	4	3 (18)	2 (67)	0	1 (33)	
	5	2 (12)	1 (50)	0	1 (50)	
Meditation	1	4 (5)	4 (100)	0	0	0.031
	2	22 (27)	9 (41)	3 (14)	10 (45)	
	3	27 (34)	7 (26)	12 (44)	8 (30)	
	4	19 (24)	7 (37)	2 (11)	10 (53)	
	5	9 (11)	4 (44)	2 (22)	3 (33)	
Eating healthy	1	10 (4)	5 (50)	2 (20)	3 (30)	0.508
	2	48 (22)	15 (31)	17 (35)	16 (33)	
	3	78 (36)	35 (45)	22 (28)	21 (27)	
	4	40 (19)	18 (45)	10 (25)	12 (30)	
	5	41 (19)	24 (59)	9 (22)	8 (20)	
Counseling/support groups	1	2 (3)	1 (50)	0	1 (50)	0.557
	2	22 (37)	5 (23)	6 (27)	11 (50)	
	3	18 (30)	10 (56)	4 (22)	4 (22)	
	4	9 (15)	3 (33)	3 (33)	3 (33)	
	5	9 (15)	4 (44)	1 (11)	4 (44)	

AA, aplastic anemia; MDS, myelodysplastic syndrome; OTC, over the counter; PNH, paroxysmal nocturnal hemoglobinuria.

a Not all patients answered each question.

b 1 = not helpful; 5 = extremely helpful.

## 4 DISCUSSION

Our findings showed that patients with MDS, AA, and PNH have severe levels of fatigue with decreased QoL. Pain and depression were mild and anxiety was moderate. The fatigue management strategies most commonly used at any frequency and perceived to be helpful were energy preservation, physical activity, and naps. The current study is among the first to report the most common fatigue management strategies used and patients’ perception of the helpfulness of these strategies in this population.

There is sparse literature covering fatigue and QoL for patients with these rare hematologic diseases, with most of the focus on QoL. A prior prospective study comparing patients’ with their physicians’ assessment of the patient's QoL showed that fatigue was not a prevalent symptom; however, physical QoL and energy levels were low.^23^ Transfusion‐dependent patients had the worst QoL scores, and multivariable analysis showed that hemoglobin levels and the presence of comorbidities were major influences on QoL. In that study, physicians underestimated the effects of QoL on their patients. The authors also noted that physicians’ judgment of the patient's well‐being must not be substituted for patient‐reported outcomes.^23^

In another report focusing on 280 high‐risk MDS patients, the three most prevalent symptoms were fatigue (92%), dyspnea (63%), and pain (55%). Patients with high levels of fatigue had greater symptom burdens and poorer QoL than those with lower levels of fatigue. In addition, patient‐reported fatigue severity was more accurate in predicting QoL than the degree of anemia.^19^

Recently, the development and validation of the quality of life in myelodysplasia scale (QUALMS) were reported.^29^ This is a 38‐item assessment tool for patients with MDS. A total of 255 patients were enrolled in an international multicenter cohort, and findings suggested that QUALMS is an important tool for assessing QoL in patients with MDS. Fatigue was ranked as the most important domain.

In patients with PNH, fatigue and decreased QoL were reported in the original eculizumab trials (TRIUMPH and SHEPHERD), and improvement in both was noted with treatment with eculizumab.^30^, ^31^ Fatigue and abdominal pain were reported as relevant issues by Meyers et al^32^ in a small study of 29 patients with PNH. Consistent with these findings, our study showed overall severe fatigue among each of the three disease subgroups. However, our cohort was fairly young, and some of these patients may have had relatively indolent disease, as indicated by their recent treatment status. This may influence fatigue levels; in an older cohort, we may expect fatigue to be even more severe. To the best of our knowledge, there are no studies describing fatigue management strategies used, frequency of use, or perceived helpfulness of these strategies in patients with these rare disease entities.

Our study has some limitations. It was an observational study of patient‐reported outcomes of three very rare diseases, with data drawn from a large international database, representing a heterogeneous sample in terms of disease type. We analyzed results from only a small percentage of the patients in the database and, and we cannot compare these data with that of nonresponders. In addition, although the database was large, with more than 17 000 patients and an international distribution, we do not know how many had recently interacted or how often they interacted with the website prior to study initiation. However, these are exceptionally rare diseases, and the success of studying these patients in a larger, more structured study is extremely difficult. In addition, although we analyzed responses from only a small fraction of potentially eligible patients, we were still able to collect valuable information concerning levels of fatigue and other common symptoms, methods of coping with fatigue, the frequency with which specific strategies were used, and patients’ perception of the helpfulness of these strategies. Most of these variables have never been reported in this population, so the current study adds valuable insight to the literature.

Furthermore, not every patient answered all questions, so there were varying numbers in the responses. However, in a survey study such as ours, this cannot be controlled, and we cannot know if it introduced bias or significantly impacted our results. However, considering the rarity of these diseases, we believe that the information we obtained gives us additional initial insight into fatigue, fatigue interventions, frequency of use of these interventions, and the perception of the helpfulness of these interventions from these patients.

Another limitation may be the subjectivity of patient‐reported outcomes. These findings cannot be validated. However, as noted in a previous study, the patient's perception of the symptom has tremendous value. 23 In fact, patient‐reported outcomes are now heavily used in studies of most symptoms. Additionally, the tools we used were previously validated, and all patients responded to the English version of the specified survey tool.

We also acknowledge the relatively low heterogeneity of the cohort with regards to race, marital status, and education; most patients described themselves as white, married, and with relatively high levels of education. The influence of these factors on the results is unknown, and our results may differ from those representing a more diverse population. We did not determine hemoglobin levels at the time the patients completed the survey tools. However, we do know that only 19% of patients reported receiving a red blood cell transfusion in the past 90 days, from which we may infer that most patients were not transfusion‐dependent. This may also indicate that factors other than hemoglobin levels could contribute to the level of fatigue experienced by these patients.

We can conclude that fatigue is an important symptom in MDS, AA, and PNH, considering that patients with all three diseases reported severe levels, and there were no significant differences among the subgroups. In addition, the strategies most patients used to cope with fatigue were preserving energy, physical activity, and naps. Patients’ perception of helpfulness of these strategies may impact their continued use. Whether these strategies are beneficial and decrease fatigue levels need to be studied further. These initial findings are important in further pursuing methods to address fatigue in patients with these very rare chronic diseases.

## CONFLICT OF INTEREST

There are no conflicts of interest to report.
